# Comparison of monocyte gene expression among patients with neurocysticercosis-associated epilepsy, Idiopathic Epilepsy and idiopathic headaches in India

**DOI:** 10.1371/journal.pntd.0005664

**Published:** 2017-06-16

**Authors:** Vasudevan Prabhakaran, Douglas A. Drevets, Govindan Ramajayam, Josephine J. Manoj, Michael P. Anderson, Jay S. Hanas, Vedantam Rajshekhar, Anna Oommen, Hélène Carabin

**Affiliations:** 1 Department of Neurological Sciences, Christian Medical College, Vellore, India; 2 Dept. of Internal Medicine, University of Oklahoma HSC, and the VA Medical Center, Oklahoma City, United States of America; 3 Dept. of Biostatistics and Epidemiology, University of Oklahoma HSC, Oklahoma City, United States of America; 4 Dept. of Biochemistry and Dept. of Surgery, University of Oklahoma HSC, Oklahoma City, United States of America; Universidad Nacional Autonoma de Mexico, MEXICO

## Abstract

**Background:**

Neurocysticercosis (NCC), a neglected tropical disease, inflicts substantial health and economic costs on people living in endemic areas such as India. Nevertheless, accurate diagnosis using brain imaging remains poorly accessible and too costly in endemic countries. The goal of this study was to test if blood monocyte gene expression could distinguish patients with NCC-associated epilepsy, from NCC-negative imaging lesion-free patients presenting with idiopathic epilepsy or idiopathic headaches.

**Methods/Principal findings:**

Patients aged 18 to 51 were recruited from the Department of Neurological Sciences, Christian Medical College and Hospital, Vellore, India, between January 2013 and October 2014. mRNA from CD14+ blood monocytes was isolated from 76 patients with NCC, 10 Recovered NCC (RNCC), 29 idiopathic epilepsy and 17 idiopathic headaches patients. A preliminary microarray analysis was performed on six NCC, six idiopathic epilepsy and four idiopathic headaches patients to identify genes differentially expressed in NCC-associated epilepsy compared with other groups. This analysis identified 1411 upregulated and 733 downregulated genes in patients with NCC compared to Idiopathic Epilepsy. Fifteen genes up-regulated in NCC patients compared with other groups were selected based on possible relevance to NCC, and analyzed by qPCR in all patients’ samples. Differential gene expression among patients was assessed using linear regression models. qPCR analysis of 15 selected genes showed generally higher gene expression among NCC patients, followed by RNCC, idiopathic headaches and Idiopathic Epilepsy. Gene expression was also generally higher among NCC patients with single cyst granulomas, followed by mixed lesions and single calcifications.

**Conclusions/Significance:**

Expression of certain genes in blood monocytes can distinguish patients with NCC-related epilepsy from patients with active Idiopathic Epilepsy and idiopathic headaches. These findings are significant because they may lead to the development of new tools to screen for and monitor NCC patients without brain imaging.

## Author summary

*Taenia solium* is a parasite normally transmitted between humans and pigs in areas with poor sanitation. Neurocysticercosis (NCC) occurs when humans are infected with larvae of *T*. *solium* that are shed with human feces and the larvae establish in the brain. NCC is often accompanied by neurological symptoms such as epilepsy. In fact, NCC causes approximately one-third of epilepsy cases in areas where *T*. *solium* is common. Unfortunately, diagnosis of NCC requires brain computerized tomography or magnetic resonance imaging, tools rarely accessible to people living where NCC is prevalent. This study tested whether genes expressed in blood monocytes, a type of white blood cell, could distinguish between people with epilepsy caused by NCC from those with epilepsy of unknown cause (idiopathic). We compared gene expression in people with NCC and epilepsy, people with idiopathic epilepsy, people cured of NCC and people without NCC or epilepsy but with headaches. We identified 15 genes which were expressed differently in the four different groups indicating that monocyte gene expression patterns in people with NCC and epilepsy are different than people with idiopathic epilepsy. These findings could lead to better understanding how humans respond to NCC and to diagnostic tests which would not require brain imaging.

## Introduction

Neurocysticercosis (NCC) is a brain infection by *Taenia solium* larvae which is a common cause of acquired epilepsy. NCC may consist of single or multiple larvae (cysts) that progress from viable vesicular to colloidal and granuloma states and finally calcify. The time required for cysts to evolve and degenerate varies from a few months to several years. NCC is responsible for nearly half of all acquired epilepsies and about one third of active epilepsies in endemic areas [1–3]. This is particularly important since epilepsy affects from 5.8 to 15.4 people per 1,000 population worldwide with a preponderance in developing countries [4]. NCC causes the largest number of disability adjusted life years among foodborne diseases [5].

Diagnosis of NCC-associated epilepsy relies on brain imaging by computerized tomography (CT) or magnetic resonance imaging (MRI), technology that is unavailable or too expensive for most people in endemic countries [6, 7]. Moreover, current blood antibody and antigen tests are not optimal for NCC diagnosis, especially for cases with low cyst numbers [8]. A reliable diagnostic test for NCC-associated epilepsy not requiring brain imaging would greatly aid its clinical management and could lead to a more comprehensive understanding of the infection.

Inflammatory host responses to degenerating cysts are related to NCC seizures development [9]. Although the relevant pathophysiologic lesions of NCC reside in the central nervous system, salient changes in peripheral blood leukocytes may be detectible. For example, Cardenas et al. detected differential expression of immune-response genes in *Taenia*-antigen stimulated peripheral blood mononuclear cells from NCC patients who did or did not demonstrate radiological response to anti-helminthic drug treatment [10]. It has also been reported that the network of monocytes and astrocytes play a role in the inflammatory response to intraparenchymal cysts of NCC [11, 12]. Furthermore, other studies show that blood monocytes produce bioactive mediators including CXCL8 (IL-8), CCL2 (MCP-1) and TNF-α, in response to stimulation with *T*. *solium* metacestode antigens [11, 13]. Moreover, emerging data show that gene expression in peripheral monocytes can be used as biomarkers to diagnose some psychiatric diseases and monitor response to therapy [14, 15]. The objective of this study was to determine the degree to which gene expression in peripheral blood monocytes could discriminate among patients with NCC-associated epilepsy, recovered NCC patients, patients with idiopathic epilepsy and patients with headaches without brain lesions.

## Methods

### Study design and sampling strategy

This cross-sectional study sequentially recruited eligible patients aged 18 to 51 years seeking care at the Department of Neurological Sciences, Christian Medical College (CMC) and Hospital, Vellore, India, between January 2013 and October 2014.

### Ethics

A research nurse explained the study objectives to eligible patients. Written consent was obtained from each patient agreeing to join the study. The study was approved by the Institutional Review Boards of Christian Medical College, Vellore, India and the University of Oklahoma HSC, Oklahoma, USA.

### Study population

Participants were categorized into four groups. Group 1 included new patients with NCC-associated epilepsy with at least one seizure in the 7 months prior to enrollment. This group was sub-divided into patients with: i) solitary cysticercus granuloma (SCG), ii) single calcified cysts (SCC) with or without edema and iii) multiple cysts at various stages of degeneration (MNCC). Group 2 included previously-diagnosed NCC patients who had recovered based on absence of seizures and brain lesions for two years or more (recovered NCC (RNCC)); Group 3 included new patients with idiopathic epilepsy with at least one seizure in the 7 months prior to enrollment, no evidence of NCC or other lesions on brain imaging and seronegative for antigens and antibodies to larval stages of *T solium*; Group 4 (idiopathic headaches) were seizure-free patients seeking consultation for headaches. These subjects had normal brain imaging, were free of seizures, head trauma, HIV, HBV HCV, and serum cysticercus antigens or antibodies. Healthy, asymptomatic subjects were not recruited as such individuals would not typically undergo serological testing for NCC. Moreover, subjecting healthy cysticercus sero-negative individuals to brain imaging would have raised ethical concerns. Patients in all groups were free of anti-inflammatory drugs (i.e. acetaminophen, ibuprofen etc) at least 7 days prior to enrollment and were not acutely ill at the time of blood sampling. NCC and RNCC patients with extra-parenchymal lesions were excluded.

The maximum age of all patients was 51 years to reduce possible confounding effects of co-existing chronic diseases. Patients were tested for HIV, HCV and HBV as clinically indicated, but not routinely tested as part of this study. Results from tests ordered during routine medical care were used to determine inclusion or exclusion. For Groups 1 and 3, patients with seizures in the past 7 months were included to maximize the potential presence of inflammatory markers circulating in the peripheral blood following a seizure.

### Definition of neurocysticercosis

The recommended NCC diagnostic criteria [16] based on brain imaging showing cystic lesions with scolex, lesions highly suggestive of NCC, or lesions compatible with NCC in combination with clinical manifestations suggestive of NCC such as epilepsy/seizures were used. All participants underwent CT or MRI of the brain. All patients in the SCG and MNCC groups and most in the SCC group had contrast enhanced studies. Images were read by one of the authors (VR). NCC lesions were categorized as described above using published recommendations [17, 18]. The MNCC group was subdivided into patients having granulomas only, calcified lesions only, or both lesion types.

### Definition of epilepsy

Epilepsy was defined according to the operational definition of the International League Against Epilepsy [19]. Thus, patients with only one epileptic seizure associated with NCC met the definition of epilepsy. All patients with Idiopathic Epilepsy had at least two lifetime seizures thereby meeting the definition.

### Measurement of exposure and current infection with *T*. *solium* cysticercosis

All patients were tested for serum antibodies to *T*. *solium* cysts by a modified enzyme-linked immunoelectrotransfer blot (EITB) [20, 21]. Additionally, all patients were tested for active infection with the AgELISA that identifies excretory/secretory products of *T*. *saginata* metacestodes in serum [22, 23].

### Blood sample collection

Blood (15–18 mL) was collected by venipuncture in heparinized BD Vacutainer tubes and processed for the isolation of monocytes. In addition, 2mL of blood was collected in clotting tubes for cyst antigen and antibody assays. Serum was separated and stored at -20°C until the assays were performed.

### Isolation and purification of monocytes

Peripheral blood mononuclear cells were isolated within one hour of blood collection over Histopaque-1077 by standard methods (Sigma-Aldrich Chemical Co, USA) and were >98% viable as measured by Trypan blue exclusion. CD14+ monocytes were isolated from mononuclear cells by immunomagnetic cell sorting using the MACS system (Miltenyi Biotec) according to the manufacturer’s recommendations. Monocyte purity was >95% as measured by flow cytometry on representative samples.

### Isolation of RNA and cDNA synthesis

RNA was extracted from monocytes with TRI Reagent following the manufacturer’s instructions (Sigma-Aldrich Chemical Co, USA) and stored at -20°C until use. RNA (10μgm) was treated with DNase to remove contaminating DNA (Ambion Life Technologies Co method, USA) and then reverse-transcribed to cDNA as described by Invitrogen Life Technologies Co, USA and stored at -80°C until used.

### Micro-array analysis

A preliminary micro-array study of a sub-group of 16 patients was performed to identify genes significantly up or down-regulated in Group 1 compared to Groups 3 and 4. cDNA samples from 16 patients (six samples each from Groups 1 and 3 and four samples from Group 4) were hybridized to U95Av2 microarrays (Affymetrix) containing 50,683 gene-probe loci and micro-array assays carried out according to the protocol of Agilent Technologies, USA. The assays were performed at Genotypic Technology Pvt. Ltd., Bangalore, India.

Initial quality check of the hybridized micro-arrays assessed the signal intensity of each gene-probe for each subject. Genes with signal intensity above background were flagged as “detected” and were further analyzed for differential expression among groups.

Genes significantly up- or down-regulated according to the p-value or Naïve Bayes classifier (see statistical analyses section below) in Group 1 compared to Groups 3 or 4 were identified. A subset of 14 genes and one pseudogene, hereafter referred to as the 15 target genes, were identified for further study based on literature review of those with biological relevance to helminthic infection, inflammatory response, and/or neurological disorders.

The genes were categorized (Table 1) based on open source databases including PubMed Gene and PANTHER [24]. Six genes encoded proteins with GTPase, GTPase activator or GTP binding activity were termed “GTP-related” and included CHN2, GBP1, GBP1P1, PLCG2, RAP1A, and TAGAP. Four genes, IL20RB, LRRFIP2, PPP2R2D, and TAX1BP1, were termed “immune-related” [25–28]. Three genes, MZB1, PECAM1, and SLC8A1, were termed “miscellaneous” and displayed protein-protein or cation binding capabilities. Two genes, FEZ2 and TOR3A, were related to “neural processes” based on Slim Molecular Function annotation [24].

**Table 1:** Peripheral blood monocyte genes initially identified by micro-array analysis selected for further study to differentiate between NCC-associated epilepsy, Idiopathic Epilepsy and idiopathic headaches.

Gene category	Gene	p-value	Bayes Classifier	FDR	Protein name	Gene Ontology Function
GTP-related	CHN2	0.0003	0.91	0.09	beta-chimerin	GTPase activator activity
	GBP1	0.0176	0.65	0.35	guanylate-binding protein 1	GTP binding/GTPase activity
	GBP1P1	0.0175	0.72	0.28	guanylate-binding protein 1 pseudogene	GTP binding/GTPase activity pseudogene
	PLCG2	0.0003	0.84	0.16	phospholipase C gamma 2	PIPLC activity
	RAP1A	0.0002	0.93	0.07	ras-related protein Rap-1A	GTP binding/GTPase activity
	TAGAP	0.0060	0.74	0.26	T-cell activation Rho GTPase-activating protein	GTPase activator activity
Immune—related	IL20RB	0.0015	0.92	0.08	IL-20 receptor subunit beta	cytokine receptor activity
	LRRFIP2	0.0001	0.99	0.01	leucine-rich repeat flightless-interacting protein 2	LRR domain binding
	PPP2R2D	0.0006	0.90	0.10	protein phosphatase 2 regulatory subunit Bdelta	protein phosphatase type 2A regulator activity
	TAX1BP1	0.0003	0.75	0.25	tax1-binding protein 1	Immune regulator
Miscellaneous	MZB1	0.0004	0.86	0.14	marginal zone B- and B1-cell-specific protein	protein binding
	PECAM1^a^ (NP113779)	0.248 (0.0006)	0.52 (0.92)	0.48 (0.08)	platelet endothelial cell adhesion molecule	protein binding
	SLC8A1	0.0008	0.96	0.04	solute carrier family 8 member A1	Calcium/sodium antiporter activity
Neural processes	FEZ2^a^ (FEZ2)	0.3260 (0.0001)	0.43 (0.91)	0.57 (0.09)	fasciculation and elongation protein zeta-2	protein binding
	TOR3A	0.0005	0.96	0.04	torsin family 3 member A	ATP binding/ATPase activity

^a^Different primer sets for these genes showed discordant results, therefore, both are reported. Names in parenthesis is the name of the gene used in micro-array

### Quantitative PCR (qPCR)

cDNA samples from all participants were subject to qPCR using the 15 target genes. The primers used for qPCR were based on probes selected from a NCBI Pubmed complete cDNA search of each target gene. qPCR reactions were carried out in a volume of 20 μl containing 1μl of 3X-diluted cDNA using SYBR Green PCR Master Mix (2X dilution) (Applied Biosystems) and consensus primers (synthesized at Sigma-Aldrich Chemicals Pvt Ltd (Bangalore, India)) (S1 Table). The reactions were performed in duplicate and each gene assayed twice. A melting curve was obtained for each PCR product after each run, confirming that the SYBR Green signal corresponded to a unique and specific amplicon.

Standard curves were generated for every qPCR run and were obtained by using serial 3-fold dilutions of a sample containing the sequence of interest. Their plots were used to convert cycle thresholds (Ct) into arbitrary quantities of initial template for a given sample. Gene expression normalized to two housekeeping genes, β2 microglobulin and 18S, was calculated by the 2^-ΔΔCt^ method [29]. Both housekeeping genes returned similar results with respect to direction (up- or down-regulation compared with comparator) and statistical significance among groups compared, but did vary in the absolute magnitude of the fold-change.

### Statistical analyses

Descriptive analyses were used to characterize the study groups. Significance among the four groups was determined using chi-square tests and Student’s t-tests.

Micro-array results were analyzed by calculating the mean log2 expression levels of each detected gene. Expression levels in Group 1 were compared with that in Groups 3 (Idiopathic Epilepsy) and 4 (idiopathic headaches) by 2 tailed Student’s t-test. The corresponding p-values were sorted from smallest to largest for all genes. Because of the exploratory nature of this analysis, a multiple correction procedure was not applied. A separate naïve Bayes classifier, similar to that of Anderson and Dubnicka [30] but modified for micro-array data, was used to compute a probability of differential expression and describe the estimated false discovery rate for each gene. This method computes areas from kernel density estimates of the log2 gene expressions for use in quantifying the probability of group affiliation for each subject. Averaging these probabilities across subjects, but within a gene, describes the “typical” probability of differential expression of that gene.

The natural logarithm (ln) of all qPCR fold changes was used to meet assumptions for parametric statistics. Significant differences of ln-transformed fold change of normalized gene expression among groups, including NCC subgroups, were determined by Student’s t-test with a p-value <0.05. Additionally, three dichotomous variables were created to characterize the type of NCC lesions: any calcification vs granuloma only, any granuloma vs calcification only, and presence of edema (yes/no). Separate analyses were conducted using the idiopathic headaches and the Idiopathic Epilepsy groups as the calibrator groups. Since using the Idiopathic Epilepsy group for calibration did not modify the conclusions regarding the comparison among NCC groups, only the results using the idiopathic headaches group are presented here. Simple linear regression models were fitted to estimate the mean difference (95% CI) in ln-transformed fold changes among all groups. Multivariable linear regression models were fitted to estimate the mean difference (95%CI) in fold changes while adjusting for potential confounders such as age, gender, time since last seizure, number of lifetime seizures and the presence of antigens or antibodies to cysticercosis among the NCC group. The linearity, normality and homoscedasticity of the studentized errors were verified visually and using the Shapiro-Wilk tests for normality and the Breush-Pagan test for homoscedasticity. All non-Bayesian analyses were conducted in Stata 14.

## Results

### Study population

Seventy-six NCC (29 SCG, 20 SCC, 27 MNCC), 10 RNCC, 29 Idiopathic Epilepsy and 17 idiopathic headaches subjects were included in the study.

Characteristics of the study population are shown in Table 2. NCC and RNCC patients showed a higher frequency of living near a pig rearing household (p = 0.01). There was a non-statistically significant larger proportion of females in the idiopathic headaches group. The NCC group had a higher percentage of patients with only one seizure in their lifetime, due to the epilepsy definition used. There was no statistically significant difference in the time since the last seizure and the type of the last seizure. Most subjects in Groups 1 and 3 had had a seizure in the past 3 months, but only two subjects had a seizure in the past 48 hours. Exclusion of these subjects did not modify the results, and hence these subjects were kept in all analyses. All subjects in Group 4 presented with headaches, but detailed data on the type, duration, and intensity of the headaches were not collected.

**Table 2:** Socio-demographic and clinical characteristics of eligible patients with different NCC lesions, RNCC, Idiopathic Epilepsy and idiopathic headaches recruited from 2013 to 2014 at the Department of Neurological Sciences, Christian Medical College (CMC) and Hospital, Vellore, India.

		Study Group						
Variable	Category	SCG	SCC	MNCC	All NCC	RNCC	Idiopathic Epilepsy	Idiopathic Headaches
**Sample size**		29	20	27	76	10	29	17
**Gender**	Male	24 (83%)	12 (60%)	18 (67%)	54 (71%)	8 (80%)	19 (66%)	7 (41%)
**Age**	Mean (SD)	30.4 (10.3)	27.1 (8.0)	31.1 (8.4)	29.8 (9.1)	30.0 (7.7)	26.9 (7.2)	34.0 (9.2)
**Schooling**	<Secondary	12 (41%)	8 (40%)	15 (56%)	35 (46%)	2 (20%)	11 (38%)	5 (29%)
≥ Secondary		17 (59%)	12 (60%)	12 (44%)	41 (54%)	8 (80%)	18 (62%)	12 (71%)
**Pork eating**	Yes	5 (17%)	3 (15%)	4 (15%)	12 (16%)	1 (10%)	2 (7%)	1 (6%)
**Living near a house that raised pigs**^a^	Yes	7 (24%)	5 (25%)	8 (30%)	20 (26%)	5 (50%)	4 (14%)	0 (0%)
**Owning pigs**	Yes	1 (3%)	0 (0%)	2 (7%)	3 (4%)	0 (0%)	0 (0%)	0 (0%)
**How often do you use the toilet to defecate?**	Never	23 (79%)	14 (70%)	18 (67%)	55 (72%)	9 (90%)	22 (76%)	16 (94%)
**Cigarette smoking**^c^	Yes	4 (14%)	6 (30%)	7 (26%)	17 (22%)	2 (20%)	4 (14%)	0 (0%)
**Paan use**^c^^,^^d^	Yes	4 (14%)	4 (20%)	8 (30%)	16 (21%)	1 (10%)	3 (10%)	0 (0%)
**Headaches**	Yes	1 (3%)	0 (0%)	2 (7%)	3 (4%)	0 (0%)	0 (0%)	17 (100%)
**EITB**^b^	Positive	11 (38%)	8 (40%)	16 (59%)	35 (46%)	3 (30%)	0 (0%)	0 (0%)
**AgELISA**^b^^,^^e^	Positive	5 (17%)	5 (25%)^§^	16 (59%)	26 (35%)^§^	1 (10%)	0 (0%)	0 (0%)
**Type of NCC lesion**	Edema	26 (90%)	4 (20%)	18 (67%)	48 (63%)	NA	NA	NA
	Only calcification	0 (0%)	20 (100%)	10 (37%)	30 (39%)	NA	NA	NA
	Only granuloma	29 (100%)	0 (0%)	11 (41%)	40 (53%)	NA	NA	NA
**Time since last seizure**	≤ 3 months	25 (86%)	17 (85%)	21 (78%)	63 (83%)	NA	21 (72%)	NA
	3–7 months	4 (14%)	3 (15%)	6 (22%)	13 (17%)	NA	8 (28%)	NA
**No of lifetime seizures**^a^^,^^b^	1	9 (31%)	0 (0%)	5 (18%)	14 (18%)	NA	0 (0%)	NA
	2–5	16 (55%)	5 (25%)	7 (26%)	28 (37%)	NA	9 (31%)	NA
	6–10	1 (3%)	1 (5%)	4 (15%)	6 (8%)	NA	2 (7%)	NA
	>10	3 (11%)	14 (70%)	11 (41%)	28 (37%)	NA	18 (62%)	NA
**Type of last seizure**	Generalized	20 (69%)	15 (75%)	19 (70%)	54 (71%)	9 (90%)	24 (83%)	NA
	Partial	6 (21%)	3 (15%)	3 (11%)	12 (16%)	1 (10%)	1 (3%)	NA
Partial then generalized		3 (10%)	2 (10%)	5 (19%)	10 (13%)	0 (0%)	4 (14%)	**NA**

SCG: single cysticercus granuloma; SCC: single calcified cyst; MNCC: multiple neurocysticercosis, NCC: neurocysticercosis; RNCC: recovered neurocysticercosis;

NA: not applicable.

^a^Statistically significant difference among Groups 1, 2, 3 and 4 using chi-square at p < .05

^b^Statistically significant difference among SCG, MNCC and SCG using chi-square at p < .05

^c^These questions were only asked to male patients. Percentages are reported among males.

^d^ “Paan” is a betel leaf with areca nut and considered a stimulant with psychoactive properties. It is chewed.

^e^AgELISA: One AgELISA test result was missing for one patient in the SCC group. Percentages are reported among those with a test result available.

There were no statistical differences among the various NCC groups with regard to age, gender, schooling, pork eating, headaches, the type of seizure and time from last seizure. However, SCG and MNCC patients were more likely to have had only one seizure than the SCC group. There was a statistically significant higher percentage of positivity by EITB and AgELISA in the MNCC patients (48% for both) compared to the SCC (11% for both) and SCG (7% for both) groups

### Micro-array analyses

Micro-array analysis found 19,778 uncompromised gene readings for all groups and showed that monocytes from patients with NCC and Idiopathic Epilepsy shared 397 up-regulated and 676 down-regulated genes compared to idiopathic headaches. Comparison of 20,739 uncompromised gene expressions of NCC with Idiopathic Epilepsy showed 1,411 up-regulated and 733 down-regulated genes specific to NCC. The probability of differential expression obtained using the naïve Bayes classifier for the up- or down-regulated genes in the latter comparison ranged from 51.3% to 98.5% and 53.3% to 98.4%, respectively.

Pathway analysis of up-regulated genes in NCC compared to Idiopathic Epilepsy revealed involvement of multiple inflammatory/immune pathways. The 10 pathways with the highest number of differentially upregulated genes included cytokine-cytokine receptor interaction, MAPK signaling, JAK-STAT signaling, Toll-like receptor signaling, and chemokine signaling (S2 Table). A pathway related to toxoplasmosis was also identified, a common protozoal brain infection. Analysis of the differentially expressed down-regulated genes also revealed pathways related to inflammation and immunity. Pathways with the greatest numbers of differentially down-regulated genes included cytokine-cytokine receptor interaction, T-cell receptor signaling, primary immunodeficiency, and MAPK signaling. These data suggest that NCC-related epilepsy elicits a monocyte gene signature that is distinct from Idiopathic Epilepsy and encompass multiple immune and inflammatory pathways.

### qPCR analysis of gene expression in NCC-associated epilepsy, Idiopathic Epilepsy and RNCC

**Fig 1:**
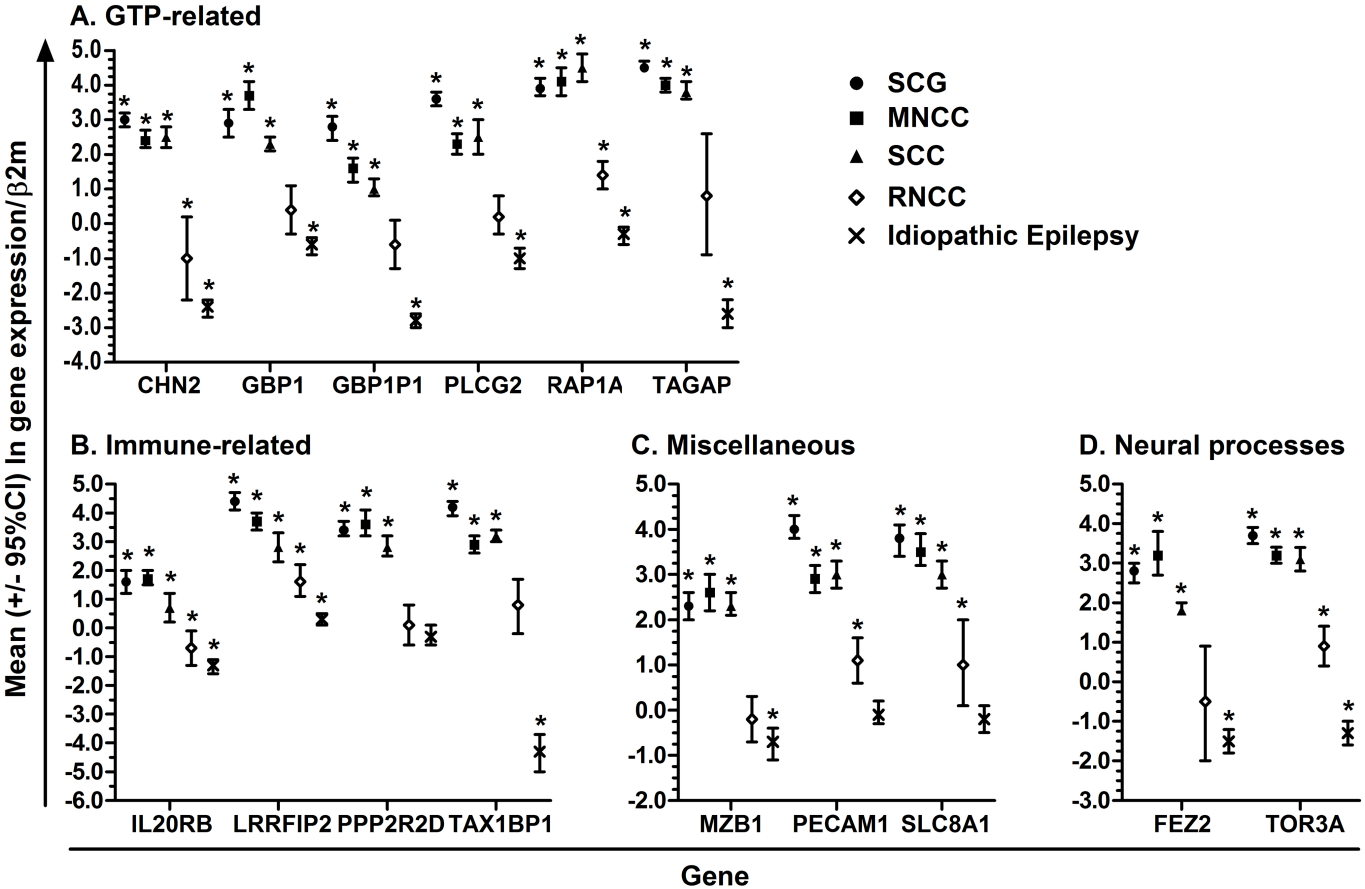
Mean (95%CI) ln-fold change in gene expression of GTP-related (Panel A), Immune-related (Panel B), Miscellaneous (Panel C) and Neural processes-related (Panel D) genes among subjects with SCG (full circle), MNCC (full square), SCC (full triangle), RNCC (open diamond) and Idiopathic Epilepsy (X) measured by qPCR, normalized to β2 microglobulin and calibrated with idiopathic headaches. *Indicates significant differences in ln-transformed fold-changes compared with idiopathic headaches using Student’s t-tests with a p-value <0.05.

Expression of each of these 15 genes was significantly increased in SCG, SCC, and MNCC compared with idiopathic headaches (Fig 1). The magnitude of expression was typically lower in RNCC patients than in patients with SCG, SCC, and MNCC and expression values of several genes in the RNCC group were not significantly different from idiopathic headaches. Fold-changes in patients with Idiopathic Epilepsy ranged from -4.3 to 0.3, with 11 genes being down-regulated compared with idiopathic headaches (Fig 1).

### Comparison of gene expression among patients with NCC and Idiopathic Epilepsy, and according to type of NCC lesion

Linear regression analysis showed all 15 genes were over-expressed in each NCC sub-group as compared with Idiopathic Epilepsy (Table 3). All genes except for IL20RB, PPP2R2D and MZB1 were more expressed in the RNCC group as compared to the Idiopathic Epilepsy group. Adjusting for age, gender, time since last seizures and total number of lifetime seizures, and sero-positivity to cysticercus antigens or antibodies did not alter these conclusions.

**Table 3:** Linear regression coefficient (95%CI) of the ln-fold change in gene expression comparing NCC and Idiopathic Epilepsy groups. β2M was used for normalization and the idiopathic headaches for calibration.

Comparison group	Idiopathic Epilepsy				MNCC	SCC	SCC
Study group	SCG	SCC	MNCC	RNCC	SCG	SCG	MNCC
**CHN2**	5.5 (5.1; 5.8)^a^	4.9 (4.5; 5.4)^a^	4.9 (4.5; 5.3)^a^	1.4 (0.9; 2.0)^a^	0.6 (0.2; 1.0)^a^	0.5 (0.1; 1.0)^a^	0 (-0.5; 0.4)
**GBP1**	3.5 (3.0; 4.0)^a^	2.9 (2.4; 3.4)^a^	4.3 (3.9; 4.8)^a^	1.0 (0.4; 1.6)^a^	-0.8 (-1.3; -0.4)^a^	0.6 (0.1; 1,1)^a^	1.4 (0.9; 2.0)^a^
**GBP1P1**	5.6 (5.1; 6.0)^a^	3.8 (3.4; 4.3)^a^	4.3 (3.9; 4.8)^a^	2.2 (1.6; 2.8)^a^	1.2 (0.8; 1.7)^a^	1.7 (1.3; 2.2)^a^	0.5 (0.02; 1.0)^a^
**PLCG2**	4.6 (4.2; 5.0)^a^	3.5 (3.1; 4.0)^a^	3.3 (2.9; 3.7)^a^	1.2 (0.7; 1.8)^a^	1.3 (0.9; 1.7)^a^	1.1 (0.6; 1.5)^a^	-0.2 (-0.7; 0.2)
**RAP1A**	4.3 (3.8; 4.7)^a^	4.8 (4.3; 5.3)^a^	4.4 (4.0; 4.8)^a^	1.7 (1.1; 2.3)^a^	-0.1 (-0.6; 0.3)	-0.6 (-1.0; -0.1)^a^	-0.4 (-0.9; 0.06)
**TAGAP**	7.2 (6.7; 7.7)^a^	6.5 (5.9; 7.0)^a^	6.7 (6.2; 7.2)^a^	3.5 (2.8; 4.1)^a^	0.5 (0; 1.0)^a^	0.7 (0.2; 1.3)^a^	0.2 (-0.3; 0.8)
**IL20RB**	2.9 (2.4; 3.3)^a^	2.1 (1.6; 2.6)^a^	3.0 (2.6; 3.5)^a^	0.6 (-0.03; 1.2)	-0.1 (-0.6; 0.3)	0.8 (0.3; 1.3)	1.0 (0.5; 1.5)^a^
**LRRFIP2**	4.1 (3.7; 4.5)^a^	2.5 (2.0; 2.9)^a^	3.4 (3.0; 3.8)^a^	1.3 (0.8; 1.9)^a^	0.7 (0.3; 1.1)^a^	1.6 (1.2; 2.1)^a^	0.9 (0.5; 1.4)^a^
**PPP2R2D**	3.7 (3.3; 4.2)^a^	3.1 (2.6; 3.6)^a^	3.9 (3.4; 4.4)^a^	0.4 (-0.2; 1.0)	-0.2 (-0.7; 0.3)	0.6 (0.1; 1.1)^a^	0.8 (0.3; 1.3)^a^
**TAX1P1**	8.5 (7.9; 9.1)^a^	7.6 (6.9; 8.2)^a^	7.3 (6.7; 7.8)^a^	5.1 (4.3; 5.9)^a^	1.2 (0.7; 1.8)^a^	0.9 (0.3; 1.6)^a^	-0.3 (-0.9; 0.3)
**MZB1**	3.1 (2.6; 3.5)^a^	3.1 (2.6; 3.5)^a^	3.3 (2.9; 3.8)^a^	0.5 (-0.1; 1.1)	-0.3 (-0.7; 0.1)	-0.01 (-0.5; 0.5)	0.3 (-0.2; 0.8)
**PECAM1**	4.1 (3.8; 4.5)^a^	3.1 (2.7; 3.5)^a^	3.0 (2.6; 3.3)^a^	1.2 (0.7; 1.7)^a^	1.1 (0.8; 1.5)^a^	1.0 (0.6; 1.4)^a^	-0.1 (-0.5; 0.3)
**SLC8A1**	4.0 (3.5; 4.4)^a^	3.2 (2.7; 3.7)^a^	3.7 (3.3; 4.2)^a^	1.2 (0,6; 1.8)^a^	0.2 (-0.2; 0.7)	0.8 (0.3; 1.3)^a^	0.6 (0.1; 1.0)^a^
**FEZ2**	4.2 (3.7; 4.8)^a^	3.3 (2.8; 3.9)	4.7 (4.2; 5.3)^a^	1.0 (0.2; 1.7)^a^	-0.5 (-1.0; 0.05)	0.9 (0.3; 1.5)^a^	1.4 (0.8; 2.0)^a^
**TOR3A**	5.0 (4.7; 5.3)^a^	4.4 (4.0; 4.7)^a^	4.4 (4.1; 4.8)^a^	2.2 (1.7; 2.6)^a^	0.6 (0.2; 0.9)^a^	0.6 (0.3; 1.0)^a^	0 (-0.3; 0.4)

^a^Indicates a significant difference at p < .05

Twelve of the 15 genes decreased significantly with the resolution of solitary cyst infections, i.e., as SCG evolved to SCC (Table 3). One exception was observed with RAP1A which was higher among those with SCC as compared to SCG.

**Fig 2:**
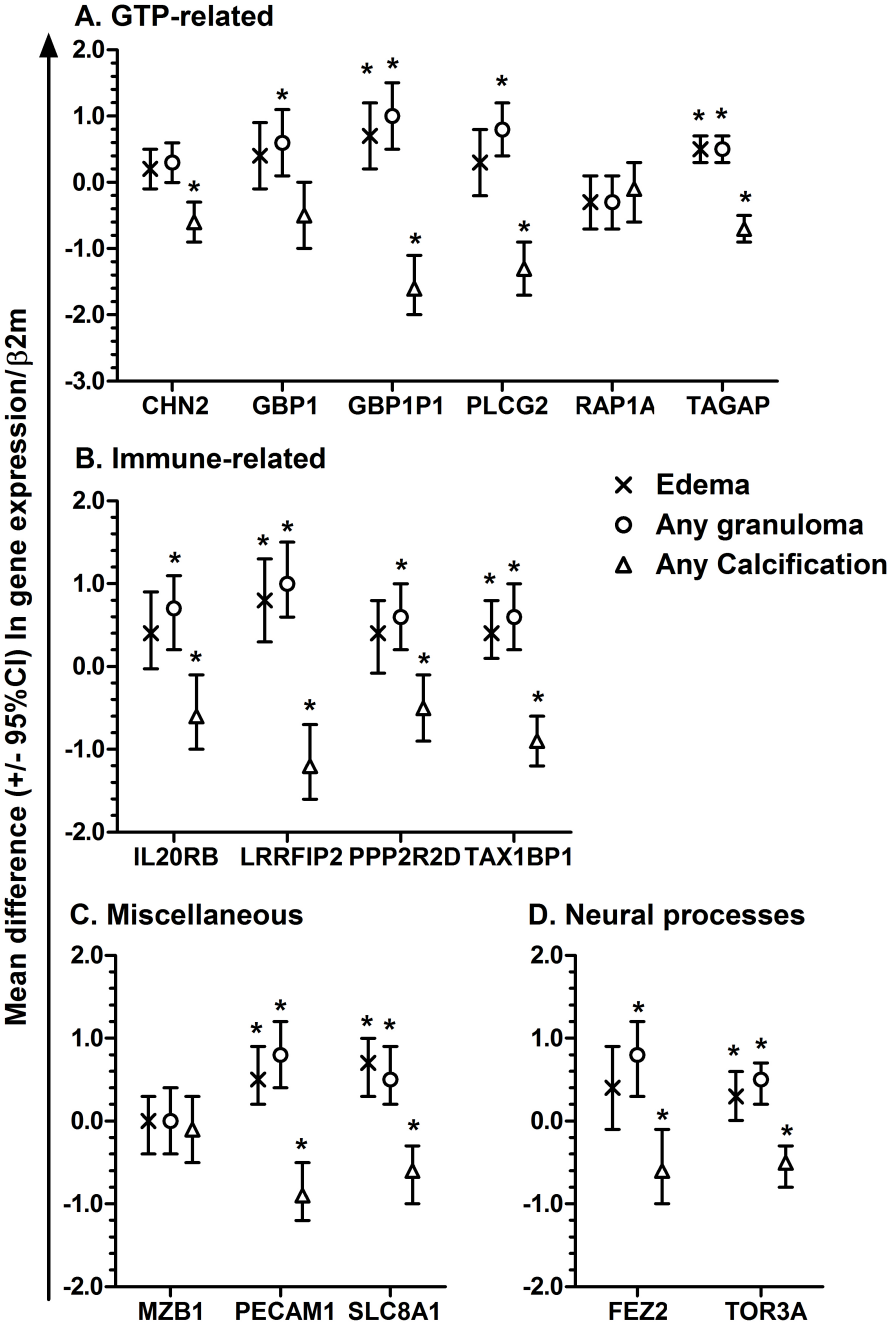
Mean differences (95%CI) in ln-fold change in gene expression of GTP-related (Panel A), Immune-related (Panel B), Miscellaneous (Panel C) and Neural processes-related (Panel D) genes among subjects with edema (X), any granuloma (open circle) and any calcification (open triangle) measured by qPCR, normalized to β2 microglobulin and calibrated with idiopathic headaches. *Indicates significant differences in ln-transformed fold-changes compared with subjects not showing that characteristic using Student’s t-tests with a p-value <0.05.

**Fig 3:**
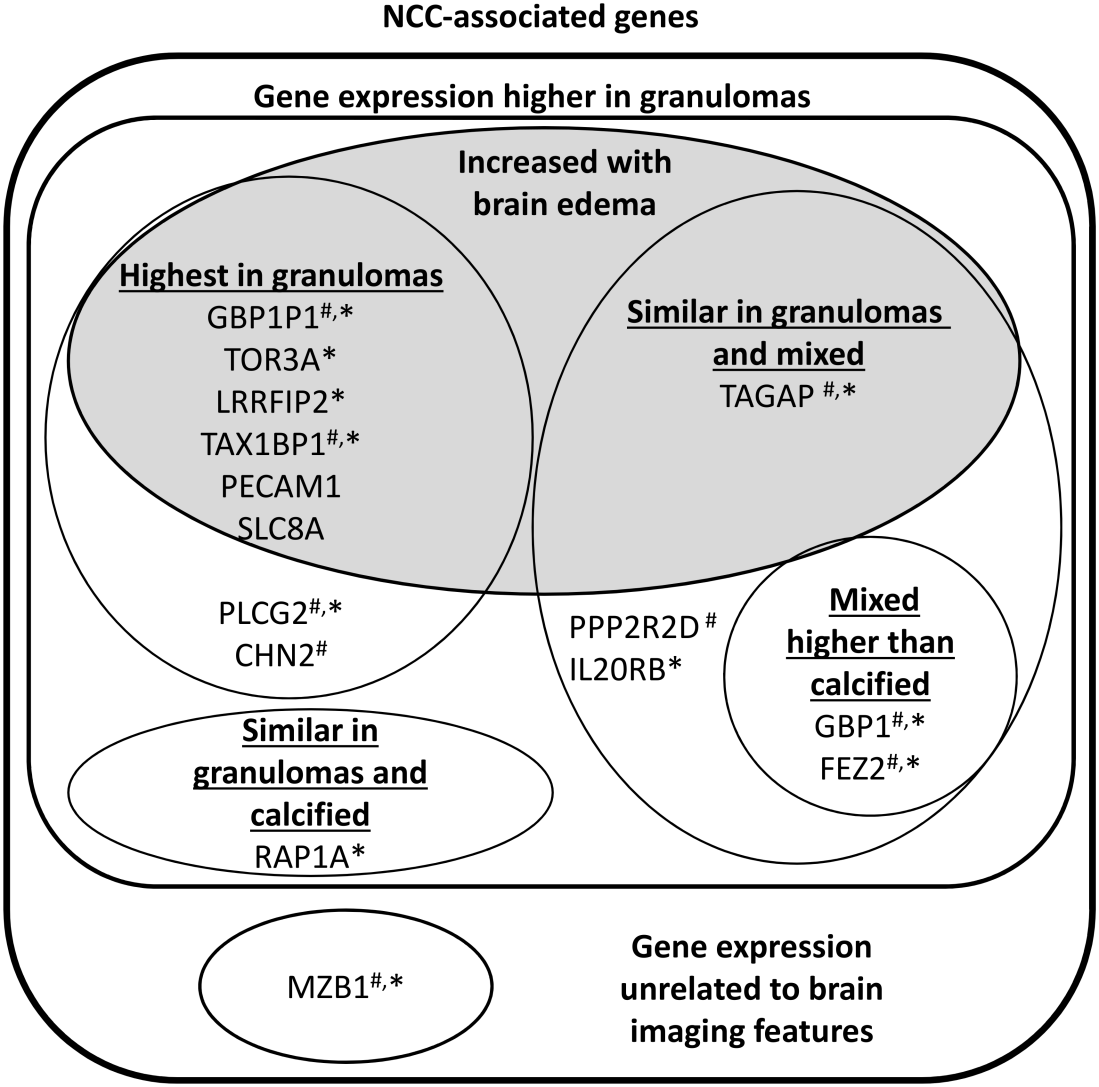
Schematic representation of monocyte genes grouped accordingly to their relationship with brain imaging features. ^#^Expression of the gene in patients with resolved NCC is comparable to its expression in normal idiopathic headaches. *Expression of the gene in patients with idiopathic epilepsy is lower than found in idiopathic headaches.

The distribution of lesions in each NCC subgroup is presented in Table 2. Twelve genes were over expressed among patients who only had granulomas as compared to patients with only calcifications (Table 4). Seven genes were over expressed in patients with edema, all of which were also over expressed among patients with any granulomas (Fig 2). RAP1A and MZB1 did not show any difference in expression according to any granuloma, any calcification or edema (Fig 2). Fig 3 summarizes associations of the gene expressions with brain lesions and edema.

**Table 4:** Linear regression coefficients (95%CI) comparing the granuloma only, calcified only, and combined (granuloma and calcified) type of NCC.

**Comparison group**	Calcification only (30 with)	Calcification only (30 with)	Mixed (6)
**Lesion**	Granuloma only (40 with)	Mixed (6)	Granuloma only (40)
**CHN2**	0.4 (0.1; 0.6)^a^	-0.2 (-0.7; 0.4)	0.5 (0.008; 1.1)^a^
**GBP1**	0.5 (-0.006; 1.0)	1.0 (0.03; 1.9)^a^	-0.5 (-1.4; 0.5)
**GBP1P1**	1.3 (0.8; 1.7)^a^	-0.7 (-1.5; 0.1)	1.9 (1.2; 2.7)^a^
**PLCG2**	1.0 (0.6; 1.4)^a^	-0.5 (-1.2; 0.2)	1.5 (0.8; 2.2)^a^
**RAP1A**	-0.2 (-0.6; 0.2)	-1.0 (-1.8; -0.2)^a^	0.8 (0.009; 1.5)^a^
**TAGAP**	0.5 (0.3; 0.8)^a^	0.2 (-0.3; 0.7)	0.3 (-0.1; 0.8)
**IL20RB**	0.7 (0.2; 1.4)^a^	0.5 (-0.3; 1.4)	0.1 (-0.7; 1.0)
**LRRFIP2**	1.2 (0.7; 1.6)^a^	0.1 (-0.8; 0.9)	1.1 (0.3; 1.9)^a^
**PPP2R2D**	0.6 (0.2; 1.0)^a^	0.6 (-0.2; 1.3)	0 (-0.7; 0.8)
**TAX1P1**	0.8 (0.5; 1.1)^a^	-0.8 (-1.4; -0.2)^a^	1.6 (1.0; 2.1)^a^
**MZB1**	0.1 (-0.3; 0.5)	-0.2 (-1.0; 0.5)	0.3 (-0.4; 1.0)
**PECAM1**	0.9 (0.5; 1.3)^a^	0.2 (-0.5; 0.9)	0.7 (0.1; 1.4)^a^
**SLC8A1**	0.6 (0.3; 1.0)^a^	-0.1 (-0.8; 0.7)	0.7 (-0.005; 1.4)
**FEZ2**	0.8 (0.3; 1.2)^a^	1.0 (0.2; 1.9)^a^	-0.3 (-1.2; 0.6)
**TOR3A**	0.5 (0.3; 0.8)^a^	0 (-0.5; 0.5)	0.5 (0.03; 1.0)^a^

^a^Indicates a significant difference at p < .05

## Discussion

Analysis of host responses in peripheral blood is one possible avenue for developing new diagnostic tools [31]. Results presented here suggest that gene expression in peripheral blood monocytes can distinguish patients with NCC-related epilepsy from patients with Idiopathic Epilepsy and from subjects negative to standard antigen and antibody assays, with normal brain imaging, no seizures and presenting with headaches. Moreover, we found that expression of 15 selected target genes in carefully selected patients with different NCC-related brain lesions including SCG, SCC, and MNCC showed differences in the magnitude of gene expression between active and recovered infections. Such differences were also observed according to the type of lesion (granuloma, calcification, edema). In general, patients with NCC showed higher magnitude of expression than did patients with recovered NCC. In addition, gene expression among these target genes was reduced as lesions resolved, i.e. as seen by the granuloma to calcification progression. Our data add to the evidence that responses in blood monocytes may be used to investigate peripheral markers that differentiate patients with and without NCC-associated epilepsy [12].

Since adjustment for age, gender, the time since the last seizure, number of lifetime seizures did not alter the conclusions and that the type of seizures, pork eating habits and headaches among the NCC group were similar, the increased gene expression in these patients compared to the idiopathic epilepsy and NCC-free headache groups may indicate expression specific to *T*. *solium* infection of the brain *per se* and not to seizures. However, the expressions may also reflect responses to brain infections in general. Increased expression of genes being due to *T*. *solium* infection of the brain and not to seizures would also be supported by their higher expression in RNCC patients compared to those with idiopathic epilepsy. Although headaches are a common symptom of NCC [32], there were large differences in the expression of target genes in patients with NCC-associated epilepsy as compared to idiopathic headaches subjects.

Results presented here agree with studies by Gupta *et al* [33] showing perilesional inflammation decreased as solitary granulomas calcified. The transition to calcification was accompanied by recovery of a breached blood brain barrier measured by reduced serum matrix metalloproteinase-9 levels. The observation that perilesional inflammation that was higher in symptomatic (seizures) compared to asymptomatic NCC patients with solitary cyst calcifications [34] may in fact be reflected by our finding of low inflammatory gene expression among RNCC patients. Together these may reflect our findings of reduced inflammatory gene expression with resolution of cysts and of low expression among RNCC patients.

There is a clear need for sensitive and accurate diagnostic tests for NCC that do not rely upon brain imaging technology and perform better than current serological tests. Results presented here support the idea that blood-based diagnostics, in particular monocyte gene expression, may be a useful tool for diagnosing and staging NCC, and perhaps also for assessing resolution. Further studies controlling for other brain infections and space occupying lesions, e.g. malignancies, are needed to establish a specific association between up-regulation of these genes and NCC.

## Supporting information

S1 Table Gene name, NCBI number, forward and reverse primers of the genes and pseudogene used for qPCR. (DOC)

S2 Table Pathways up-regulated in subjects with NCC associated seizures compared to the idiopathic epilepsy group. (DOC)

S1 Dataset Uncompromised micro-array data comparing the idiopathic headache group (columns G-J) to the idiopathic epilepsy group (columns L-Q) to the NCC group (columns U-Z). Please refer to the readme tab for more details.
(XLSX)

S2 Dataset Uncompromised micro-array data comparing the idiopathic epilepsy group (columns G-L) to the NCC group (columns N-S). Please refer to the readme tab for more details.
(XLSX)

S3 Dataset Fold change in qPCR using β2M for normalization and the idiopathic headache group for calibration. Please refer to the readme tab for more details.
(XLSX)
