# Supplementary material for: Comparison of monocyte gene expression among patients with neurocysticercosis-associated epilepsy, Idiopathic Epilepsy and idiopathic headaches in India
Source: PLoS Negl Trop Dis. 2017 Jun 16;11(6):e0005664. doi: 10.1371/journal.pntd.0005664 (PMC5489221; doi:10.1371/journal.pntd.0005664)
Supplement: S1 Table — (DOC) [file pntd.0005664.s001.doc]

**S1 Table. Gene name, NCBI number, forward and reverse primers of the genes and pseudogene used for qPCR**

| **Gene** | **NCBI** | **Forward Primer** | **Reverse Primer** |
| --- | --- | --- | --- |
| LRRFIP2 | NM_006309 | AGGTCTTGGAGTCAGCAGGAGA | CACTGTGCCATCATTCCTGGAG |
| FEZ2 | NM_005102 | GTGATGCCTGTAGACTGGAAGTC | CACAGGAGACGATGATTGAGTGC |
| RAP1A | NM_002884 | ACTTACAGGACCTGAGGGAACAG | CCTGCTCTTTGCCAACTACTCG |
| CHN2 | NM_004067 | CATCCTTAGAGAAAGCCAGCGG | CGACTCAAACCTCTTCTCACCC |
| TOR3A | NM_022371 | GGTGGTCCTAAAGTTGCTCAAGG | CAGGTTTTCCTTCACAAGACGGC |
| PECAM1 | NM_000442 | AAGTGGAGTCCAGCCGCATATC | ATGGAGCAGGACAGGTTCAGTC |
| IL20RB | NM_144717 | GCCATACAACCTTCGTGTCAGG | ACCAGGTGGAAGCCATCTTTGG |
| PPP2R2D | NM_018461 | CGTGTTCGTCTACAGCAGTAGC | CTGAGAAGAAGGACCTACTGCTG |
| SLC8A1 | NM_021097.2 | GCCTGGAGCATCTTTGCCA | CCCAAGCGAACACAACACAG |
| TAGAP | NM_054114 | ATGACTCCCTGGAGCACACTGA | CTGTTGGATTCCACATCAGGGTC |
| TAX1BP1 | NM_006024 | GTTAGCTGATGCAGTGGCAGAAC | CAGCCATCTGAAGACGGAGTTTC |
| PLCG2 | NM_002661 | AGCGACTCCTATGCCATCACCT | AACTGACGAGCTCCACCAGACT |
| MZB1 | NM_016459 | TGAGCGAGTTGGTCTACACGGA | ACCATCACGCTGATGCTTGGCT |
| GBP1 | NM_002053 | TAGCAGACTTCTGTTCCTACATCT | CCACTGCTGATGGCATTGACGT |
| GBP1P1 | NR_003133 | GACTGAGAAGATGGAGAGCGAC | GTAAGCCTGTGATCGGGGAGAA |
| B2M | NM_004048.2 | AGATGAGTATGCCTGCCGTG | TCATCCAATCCAAATGCGGC |
| 18S rNA | NR_003286.2 | GGAGTATGGTTGCAAAGCTGA | ATCTGTCAATCCTGTCCGTGT |
