# Supplementary material for: Comparison of monocyte gene expression among patients with neurocysticercosis-associated epilepsy, Idiopathic Epilepsy and idiopathic headaches in India
Source: PLoS Negl Trop Dis. 2017 Jun 16;11(6):e0005664. doi: 10.1371/journal.pntd.0005664 (PMC5489221; doi:10.1371/journal.pntd.0005664)
Supplement: S2 Table — (DOC) [file pntd.0005664.s002.doc]

**S2 Table.** Pathways up-regulated in subjects with NCC associated seizures compared to the idiopathic epilepsy group

| **Pathway Name** | **KEGG ID** | **Genes** | **No. of Genes** |
| --- | --- | --- | --- |
| Metabolic pathways  [2238] | hsa01100 | HSD11B1,DPYD,NT5C3,CD38,ACAT2,ADA,DHFR,PTGES,HAO1,UROS,BCKDHB,KHK,KMO,AK4,ETNK1,DLST,CSGALNACT2,PTGS2,HK2,CES1,MAT2B,B4GALT1,PIP5K1B,MAN1A1,INPP4A,FLJ36848,AANAT,UAP1,INPPL1,POLR1D,ATP6V1C1,H6PD,BCAT1,POLR2E,POLR3G,PI4K2B,ITPK1,ATP6V1B2,LSS,PIGA,LAP3,SMS,ADPGK,SGMS2,SGMS1,LPCAT2,GLUL,MGAM,LPCAT1,UPB1,GK,PI4KA,ACSL1,PDXK,CMAS,CYP3A7,UXS1,EXT1,ALG13,ARSB,IL4I1,MCCC2,AGPAT4,CYP27B1,AOC3,HMGCS1,MTHFD2,ATP6AP1,GLYCTK,SAT1,KYNU,AFMID,GALNT3,TKTL2,PAFAH1B1,IDO2,IDO1,EPT1,MGAT1,GCH1,CMPK2,CEPT1,B3GNT5,MGLL,NDUFB3,NDUFB5 | 86 |
| Cytokine-cytokine receptor interaction [550] | hsa04060 | IL1RAP,CSF3,CSF2,IL18,IL15,IL1B,IL1A,IL29,CCL4,IL6ST,CD40,CCR5,CCL3L3,CXCL1,CXCL3,CXCL2,CXCL9,IL10RA,OSM,TNFRSF9,TNF,IFNB1,IL23A,IFNA7,IFNA4,IFNA8,CCL26,CCL23,CCL20,CCL19,CCL16,CCL18,FLT1,LIF,BMP2,LTBR,TNFRSF10B,LEPROT,IFNGR2,INHBA,IL12A,IL12B,IL6,IL8,CXCL11,CXCL10,CXCL14,CXCL16,TNFSF8,PRLR,IFNA14,FAS,TNFSF15,TNFSF10,IL20RB,ACVR2A | 56 |
| Pathways in cancer [652] | hsa05200 | NRAS,CDKN2B,HIF1A,PTK2,E2F3,STAT5A,PIAS2,BID,TFG,FGFR2,BRCA2,RALGDS,PTGS2,CTNNB1,CTNNA1,CCNE2,CCND1,PML,TRAF1,TRAF3,CDK2,NCOA4,BMP2,NFKB1,NFKB2,FGF18,FGF11,RUNX1,HSP90AB1,HSP90AA1,DAPK1,CRK,MAPK9,IL6,IL8,WNT7B,CYCS,PPARD,BIRC3,BIRC2,PIK3R5,FAS,RASSF5,CDC42,LAMB3,LAMB1,LAMA2,STAT1,FZD4,STAT3,TCF7L2 | 51 |
| Toxoplasmosis [264] | hsa05145 | HLA-DRA,NFKBIB,CD40,CCR5,IL10RA,PPIF,TNF,HLA-DPA1,HSPA1A,HSPA1B,HLA-DPB1,MAP2K3,TAB2,NFKB1,SOCS1,IFNGR2,MAPK9,LDLR,IL12A,IL12B,HLA-DQB2,CYCS,MYD88,BIRC3,BIRC2,PIK3R5,HLA-DRB5,HSPA2,HSPA6,HSPA8,JAK2,LAMB3,LAMB1,LAMA2,STAT1,STAT3,TLR2 | 37 |
| MAPK signaling pathway [544] | hsa04010 | MAP3K4,MAP3K8,TAOK3,DUSP5,NRAS,DUSP8,IL1B,IL1A,ATF2,FGFR2,TNF,MAP3K2,HSPA1A,HSPA1B,MAP2K3,DDIT3,TAB2,RAPGEF2,NFKB1,NFKB2,GADD45B,GADD45A,FGF18,FGF11,CRK,MAPK6,MAPK9,PPP3CA,RELB,MAPKAPK2,FAS,CDC42,HSPA2,HSPA6,HSPA8,MAP4K3 | 36 |
| Jak-STAT signaling pathway [310] | hsa04630 | CSF3,CSF2,IL15,IL29,IL6ST,STAT5A,PIAS2,IL10RA,OSM,IFNB1,IL23A,IFNA7,IFNA4,IFNA8,CCND1,LIF,LEPROT,SOCS3,SOCS1,IFNGR2,IL12A,IL12B,IL6,PIK3R5,IRF9,PRLR,IFNA14,JAK2,IL20RB,STAT1,STAT3,STAT2,SPRED2 | 33 |
| Toll-like receptor signaling pathway [204] | hsa04620 | MAP3K8,SPP1,TICAM1,IL1B,CCL4,CD40,CXCL9,CD80,TNF,IFNB1,IFNA7,IFNA4,IFNA8,MAP2K3,TRAF3,TAB2,NFKB1,MAPK9,IL12A,IL12B,IL6,IL8,CXCL11,CXCL10,MYD88,PIK3R5,IRF7,IFNA14,STAT1,TBK1,TLR2,TLR7,TLR8 | 33 |
| Chemokine signaling pathway [378] | hsa04062 | NFKBIB,NRAS,CCL4,PTK2,CCR5,CCL3L3,CXCL1,CXCL3,CXCL2,CXCL9,FOXO3,CCL26,CCL23,CCL20,CCL19,CCL16,CCL18,VAV2,HCK,NFKB1,CRK,IL8,CXCL11,CXCL10,CXCL14,CXCL16,PIK3R5,ROCK2,CDC42,JAK2,STAT1,STAT3,STAT2 | 33 |
| Hepatitis C [270] | hsa05160 | EIF2AK2,EIF2AK3,TICAM1,NRAS,PIAS2,OAS3,OAS1,OAS2,NR1H3,TNF,IFNB1,IFNA7,IFNA4,IFNA8,TRAF3,IFIT1,NFKB1,SOCS3,MAPK9,LDLR,IL8,PIK3R5,IRF9,IRF7,IRF1,IFNA14,PPP2CA,DDX58,STAT1,STAT3,STAT2,TBK1 | 32 |
| Osteoclast differentiation [256] | hsa04380 | BTK,IL1B,IL1A,FCGR3A,FCGR2C,TNF,IFNB1,TAB2,GAB2,LILRA3,LILRB5,LILRB4,NFKB1,NFKB2,SOCS3,SOCS1,IFNGR2,MAPK9,PPP3CA,RELB,CYBB,PIK3R5,IRF9,SIRPA,FOSL2,LCP2,CYLD,STAT1,STAT2 | 29 |
